# Preventing breast cancer now by acting on what we already know

**DOI:** 10.1038/npjbcancer.2015.9

**Published:** 2015-07-22

**Authors:** Graham A Colditz, Kari Bohlke

**Affiliations:** 1 Division of Public Health Sciences, Department of Surgery, Washington University in St Louis, St Louis, MO, USA; 2 Siteman Cancer Center, Washington University in St Louis, St Louis, MO, USA

## Abstract

The age-specific rate of breast cancer rises rapidly through premenopausal years and significantly more slowly after menopause. Reproductive factors affect cell proliferation and the accumulation of genetic changes. Lifetime risk of breast cancer is linearly related to the length of the interval from menarche to first birth. Lifestyle changes that accompany industrialization, together with shifting reproductive patterns, drive up incidence rates. Prevention must begin early in the life as almost one-quarter of cases are diagnosed before age 50 in high-income countries. This requires greater emphasis on prevention across the life course to address the global burden of breast cancer.

The burden of breast cancer is worldwide and growing. In 2012, nearly 1.7 million new cases of breast cancer were diagnosed worldwide, accounting for 25% of all new cancer cases in women.^1^ The incidence of the disease has increased sharply in low- and middle-income countries, a trend expected to continue as economic development creates lifestyle factors that heighten breast cancer risk for women in those countries. Moreover, low- and middle-income nations cannot afford the costs of widespread technology-based medical care for their populations, whereas in wealthy nations, projected expenses for clinical treatment are soaring.^2^

It is time to place more emphasis on breast cancer prevention, that is, lowering the risk or reducing the incidence of new cases. Evidence from a wide range of studies—randomized trials, epidemiological, animal—has identified specific lifestyle and behavioral factors that affect breast cancer risk. Researchers also now better understand that breast cancer risk develops early in life and accumulates across a woman’s entire lifespan. This article discusses specific actions that women and their physicians can take to reduce breast cancer risk, and argues that, depending on when in her lifespan a woman integrates risk-reduction behaviors, as much as 50–70% of breast cancer can be prevented through primary prevention.^3^

What is heightening the risk of breast cancer? It is the interplay of (1) increases in hormonal drivers, (2) the changes in women’s height growth rate and reproductive patterns, and (3) lifestyle changes arising from economic development. These are not the only modifiable factors that affect breast cancer risk (exposure to ionizing radiation, for example, also increases risk),^4^ but they are among the most common. Let us start with hormones. There is very strong evidence that hormones such as estrogen and progesterone are major drivers of breast cancer growth. For example, combination estrogen plus progestin therapy has been classified as a carcinogen based on laboratory, epidemiologic, and randomized trial evidence.^5^ And data from randomized trials show that selective estrogen receptor modulators and aromatase inhibitors significantly reduce breast cancer incidence.^6^ These hormones, whether endogenous or exogenous, promote tumor growth.

Second, recent task force reports on breast cancer prevention from the US National Institutes of Environmental Health Sciences jointly with the National Cancer Institute^7^ and the Institute of Medicine^8^ urge the nation to focus on risk factors at time periods in a woman’s life that are important in the etiology of breast cancer. These time periods begin in childhood, before and during first breast development, and before the full differentiation of breast tissue that occurs with the birth of a woman’s first baby.^9^

Breast cancer incidence models show breast tissue aging or the underlying rate at which the carcinogenic process is taking place occurs most rapidly from menarche to the first birth with cyclical production of estrogen and progesterone during each menstrual cycle with increased mitotic rate during the luteal phase of the cycle.^10,11^ After menarche, cell proliferation decreases with age through premenopausal years.^10^ The rate of breast cancer increases rapidly up to menopause and then far more slowly after menopause.^10^ Early menopause (natural or due to surgical removal of ovaries) reduces subsequent risk of breast cancer.^10–13^

Third, childhood exposure to three factors—excess energy intake leading to weight gain and obesity, high animal protein intake and low intake of fruits, vegetables, and whole grains, and little or no physical activity—influences both onset of menarche and height growth rate.^14^ This is when breast tissue is most susceptible to carcinogenesis. Height is related to increased risk of breast cancer before and after menopause,^15^ and reflects nutrition intakes through childhood. Controlled trials show milk intake, for example, increases height growth rate.^16^ A prospective study of childhood diet and growth shows higher vegetable protein intake at ages 3–5 is related to slower height growth rate and later age at menarche, whereas higher animal protein intake is related to increased peak height growth velocity.^14^ In a prospective analysis of weight and height from school records of over 117,000 women in Denmark, height growth rate from 8 to 14 years of age independently predicted risk for beast cancer, accounting for 15% of the population of burden of breast cancer.^17^ Higher peak height growth velocity is positively related to the risk of premenopausal and postmenopausal breast cancer^18^ indicating that growth rate, independent of age at menarche, has a direct influence on lifelong breast cancer risk.^17,18^ China Health and Nutrition Survey data show that among ages 2–18 over the 20-year period from 1991 to 2011, a marked decrease in intake of legumes (22% decrease) and coarse grains (71% decrease) with an increase in animal-source foods (50% increase).^19^ Yet evidence supports higher fiber intake during adolescence protecting against benign breast disease and invasive breast cancer.^20–22^

These three factors plus alcohol have dramatically affected both the amount of hormones and the length of time women’s bodies are exposed to them. Beginning three centuries ago in Europe, then in the United States, economic development spurred increases in these factors that have driven these endogenous hormonal exposures within women to new levels.^23^ For example, in traditional rural life with menarche at 17, a typical woman had perhaps 26 menstrual cycles (±2 years) between menarche and the birth of her first baby. Now, with earlier menarche at, say, age 12 and a much later age of first birth (e.g., 30 years),^24^ a woman has about 234 cycles (18 years×13 cycles/year), before the full cellular differentiation that accompanies breast development during first pregnancy; this is an increase of more than 200 cycles compared with pre-industrialization reproductive patterns. This means that she is exposed to over 200 additional luteal phase proliferative cycles of breast cell growth and the potential to accumulate genetic alterations.^10^

Breast cancer in Asia over the past 50 years illustrates the impact of these factors, highlighting how changes within a population can occur, and very quickly. In Korea, age at menarche decreased from an average of 16.9 among women born in 1920–1924 to 13.8 among women born in 1980–1985 (ref. 25); fertility decreased from an average of 6 births per woman in 1960 to 5 in 1970 to 1.23 in 2010 (ref. 26) and age at first birth increased to 30, by 2010, on average.^27^ In Singapore, Hong Kong, and China, similar changes have occurred.^28^ In association with these societal changes, the incidence of breast cancer in Korea for women of age 45–49 has tripled from 45 cases/100,000 among women in born 1943–1945 (ref. 10) to 140 cases/100,000 among women born in 1960–1962 (ref. 29). China has followed suit.^30^

The how and when of breast cancer prevention. It is neither desirable nor feasible to shift reproductive patterns back to pre-industrial levels. However, the nation can support women in tackling the same four factors that originally caused shifts in societal reproductive patterns. Excess energy intake that leads to weight gain and obesity; high animal protein intake and low intake of fruits, vegetable, and whole grains; little or no physical activity; and excess alcohol consumption create a toxic physiologic context that is directly related to increased breast cancer risk at any age.^31,32^ Women in the United States who followed the healthy lifestyle had significantly lower incidence of new breast cancers (20% few cases) than those who did not, after controlling for all other risk factors, education, and race/ethnicity.^31^

Timing of prevention therefore matters. Because 22% of breast cancer is diagnosed in premenopausal women^33^ and is often more aggressive than cancers diagnosed in postmenopausal women, it makes sense to start prevention early in life when it can have maximum impact. For example, prevention begun in childhood and continuing through adolescence and early adult years can reduce development of premalignant or intermediate lesions that are on the pathway to breast cancer.^21,34^ The evidence for this comes from prospective data recorded in childhood, adolescence, and early adult years and includes evidence for alcohol, a known breast carcinogen. Women who avoided alcohol intake through adolescence and early adult years have significantly lower incidence of premalignant and invasive breast cancers.^34^

Recommendations for the reduction in breast cancer burden from the U.S. Institute of Medicine,^8^ the World Cancer Research Fund/American Institute for Cancer Research,^35^ and the American Cancer Society,^36^ when applied across the life course, generate the following potential reductions in breast cancer burden (see Table 1) to illustrate the potential benefit of beginning prevention early. A large body of epidemiologic evidence suggests that up to 68% of breast cancer could potentially be prevented by efforts that begin in childhood and adolescence; in contrast, if prevention were delayed until age 50, 22% of cancers would already be diagnosed, and a smaller percentage of all cancers (up to 50%) would be potentially preventable.^3^

Can adopting these changes really make a difference? There is precedent in the case of combination estrogen plus progestin hormone therapy use. Following the publication of results from the Women’s Health Initiative,^37^ a randomized trial that confirmed previous epidemiologic data showing increasing risk for breast cancer with increasing duration of use of combination estrogen plus progestin, population and individual level data showed a rapid decrease in use of combination estrogen plus progestin by women around the world, and incidence of breast cancer in women 50 years and older decreased.^38,39^ On the basis of these epidemiologic and randomized trial data, the International Agency for Research on Cancer classified combination estrogen plus progestin as a breast carcinogen.^5^ Rates of breast cancer in a cohort of women undergoing mammographic screening declined by 5% per year from 2000 to 2003 (ref. 38). Thus, changes in behavior can have positive effects on individual women and the aggregate of those effects can have a societal impact.

Building awareness of the potential for prevention works. Similar to the scientific evidence and prevention messaging that led to the drop in the use of estrogen plus progestin therapy, another national effort to reduce and eliminate tobacco use has had significant impact on the health of women and men.^40^ Trials show that prevention works.^41^

Building on the Institute of Medicine report, one research prevention priority is to better understand the lifestyle factors (diet patterns, exercise, etc.) in childhood and in the critical window from menarche to first pregnancy that offer the greatest protections.^8^ Because breast cancer risk gathers across a woman’s lifetime, the scientific focus must move away from a concentration on exposures immediately before diagnosis.^8^ In our view, and that of Vogelstein,^42^ achieving the full promise of breast cancer prevention calls for government and philanthropic organizations to make a much greater allocation of resources to prevention; that is, improving prevention messages and understanding for women, their daughters, and mothers. This requires full implementation of established behavioral and lifestyle strategies for cancer prevention, coupled with research that addresses risk factors and how prevention strategies can be tailored to each phase of a woman’s life. The nation knows how to do this.

Meeting this challenge will require increasing the proportion of breast cancer research that is devoted to prevention. Review of budget allocations to breast cancer research shows that at most 7% of all National Institutes of Health breast cancer research projects (not dollars) focus on breast cancer prevention, whereas only 1% of dollar expenditures in the U.S. Department of Defense Breast Cancer Research Program is allocated to research on prevention.^7^ Given this lopsidedness of resources, surely some reallocation of focus and funding is possible. Making prevention happen requires not just a plan, but the political will to acquire and sustain the resources needed to deliver breast cancer prevention across all sectors will be necessary to implement and sustain prevention with the long-term outcomes benefiting women worldwide.

Implementing a breast cancer prevention plan involves the systematic integration of evidence-based messages and strategies into women’s everyday lives. Evidence-based strategies show that installing bikeways and sidewalks increase exercise, changes in diet reduces weight gain in children,^41^ and increasing excise taxes on alcohol and tobacco products reduce consumption and lowering risk of cancer and risk of many other chronic diseases.^43^ We must put these cancer-reducing behaviors into practice, including concerted action from communities, policy makers, schools, families, and individuals alike.^44^ The fact that the same four factors are implicated not only in breast cancer but also in other cancers, heart disease, and diabetes means that there can be economies in the prevention outreach and engagement. Making these appropriate for women at differing ages is amongst the highest priorities.

Also informing prevention, impressive progress has been made in identifying genetic factors that increase breast cancer risk (e.g., mutations in BRCA1/2 and rare variants in other genes) and in developing new treatments. However, the prevention treatments available today for BRCA1/2 carriers (risk-reducing mastectomy and bilateral salpingo-oophorectomy and chemoprevention) would eliminate only 5% of breast cancers worldwide and would primarily benefit women in high-income countries.^45,46^ This subpopulation must be identified, limiting applicability. These treatments would do little to stem the increasing incidence of breast cancer globally.

If we act and act now, shifting the balance and focus to earlier life, supported by additional resources devoted to implementing prevention, bringing messages and bolstering lifestyle and risk-reduction behaviors during the critical time points in life, we stand a good chance of significantly reducing the burden of breast cancer now and for future generations.

## Figures and Tables

**Table 1:** Population prevention strategies for breast cancer

*Recommended risk-reduction strategies*	*Percentage of total breast cancers preventable in the United States (%)^a^*
Establish and maintain childhood dietary intake of vegetables, fruits, and whole grains, and reduce animal protein^3,20,35,36^	3
Increase and maintain physical activity through childhood, adolescence, and adult years^35,36^	11
Avoid and reduce weight gain during the adult years^35,36^	25–32
Achieve a sustained 10% weight loss among postmenopausal women^8^	25
Reduce or eliminate alcohol intake between menarche and first birth^35,36^	3
Reduce or eliminate alcohol intake among adult women throughout the life course^35,36^	3
Continue to reduce use of estrogen plus progestin hormone therapy (a known carcinogen)^8^	3
For high-risk women, increase access to and use of drugs targeted to reduce breast cancer risk (e.g., Tamoxifen)^6,8^	11

a Reduction in breast cancer cases estimated from population prevalence of exposure and magnitude of observed associations to estimate population attributable risk.
